# Extracellular Matrix-Specific Platelet Activation Leads to a Differential Translational Response and Protein De Novo Synthesis in Human Platelets

**DOI:** 10.3390/ijms21218155

**Published:** 2020-10-31

**Authors:** Bjoern F. Kraemer, Marc Geimer, Mirita Franz-Wachtel, Tobias Lamkemeyer, Hanna Mannell, Stephan Lindemann

**Affiliations:** 1 Medizinische Klinik und Poliklinik I, Klinikum der Universität München, Marchioninistrasse 15, 81377 Munich, Germany; bjoern.kraemer@klinik-ebe.de; 2 Klinik für Anästhesie, Intensiv- und Notfallmedizin, Westpfalz Klinikum Kaiserslautern, Hellmut-Hartert Str. 1, 67655 Kaiserslautern, Germany; mgeimer@westpfalz-klinikum.de; 3 Proteasome Center Tuebingen, University of Tuebingen, Auf der Morgenstelle 15, 72076 Tübingen, Germany; mirita.franz@uni-tuebingen.de; 4 Cluster of Excellence Cologne (CEDAD), Mass Spectrometry Facility at the Institute for Genetics, University of Köln, Josef-Stelzmann-Str. 26, 50931 Köln, Germany; tobias_lamkemeyer@yahoo.de; 5 Doctoral Programme of Clinical Pharmacy, University Hospital, Ludwig-Maximilians-University, Marchioninistr. 27, 81377 Munich, Germany; Hanna.Mannell@med.uni-muenchen.de; 6 Institute of Cardiovascular Physiology and Pathophysiology Biomedical Center, Ludwig-Maximilians-University, Großhaderner Str. 9, 82152 Planegg, Germany; 7 Philipps Universität Marburg, FB 20-Medizin, Baldingerstraße, 35032 Marburg, Germany; 8 Klinikum Warburg, Medizinische Klinik II, Hüffertstr. 50, 34414 Warburg, Germany; 9 Medizinische Klinik und Poliklinik III, Otfried-Muller-Str. 10, Universitätsklinikum Tübingen, 72076 Tübingen, Germany

**Keywords:** platelets, translation, extracellular matrix, differential protein expression, cytoskeleton

## Abstract

Platelets are exposed to extracellular matrix (ECM) proteins like collagen and laminin and to fibrinogen during acute vascular events. However, beyond hemostasis, platelets have the important capacity to migrate on ECM surfaces, but the translational response of platelets to different extracellular matrix stimuli is still not fully characterized. Using 2D-gel electrophoresis, confocal microscopy, polysome analysis and protein sequencing by mass spectrometry, we demonstrate that platelets show a differential expression profile of newly synthesized proteins on laminin, collagen or fibrinogen. In this context, we observed a characteristic, ECM-dependent translocation phenotype of translation initiation factor eIF4E to the ribosomal site. eIF4E accumulated in polysomes with increased binding of mRNA and co-localization with vinculin, leading to de novo synthesis of important cytoskeletal regulator proteins. As the first study, we included a proteome analysis of laminin-adherent platelets and interestingly identified upregulation of essentially important proteins that mediate cytoskeletal regulation and mobility in platelets, such as filamin A, talin, vinculin, gelsolin, coronin or kindlin-3. In summary, we demonstrate that platelet activation with extracellular matrix proteins results in a distinct stimulus-specific translational response of platelets that will help to improve our understanding of the regulation of platelet mobility and migration.

## 1. Introduction

Platelets are essential for cell-mediated hemostasis and activation of platelets results in a fast and effective formation of platelet aggregates and blood clots. This is especially important in pathophysiological events like myocardial infarction and stroke, where platelets are exposed to laminin, a component of the basal lamina, and collagen, the predominant extracellular matrix protein. In this process, platelets undergo remarkable transformation with rearrangement of the cytoskeleton that requires a fast and well-coordinated regulation of cytoskeletal proteins. Despite the vital function of platelet-mediated hemostasis and clot formation, uncontrolled platelet activation during acute vascular events is the most feared aggravating factor for devastating complications during myocardial infarction and stroke. Therefore, a detailed knowledge about activation pathways, intracellular signaling and metabolic processes under different environmental conditions is of utmost importance for targeted antiplatelet therapy. Previous research has shown that platelets possess the capability to synthesize proteins de novo despite lack of a nucleus [1]. Although the endpoint of platelet activation seemed similar for all platelet activators, previous research demonstrated that different platelet activators induce a differential proteome [2,3]. Besides classical functions of hemostasis, previous work from our group and others has discovered that platelets are able to perform a much more complex and dynamic cytoskeletal rearrangement that enables platelets to migrate [4,5,6,7,8,9,10,11]. These studies demonstrated that platelets can migrate under flow conditions, towards a cytokine gradient or along bacterial trails, and that morphology and phenotype of migrating platelets differs depending on the underlying extracellular matrix surface [6,7,8]. Migration allows platelets for instance to transmigrate into the vessel wall [7,9] and to collect and phagocytose bacterial pathogens [6]. Overall, the required complex cytoskeletal adaptation and rearrangement during migration appears to involve ion channel regulation [6,11], post-translational protein modification such as phosphorylation [12], protein cleavage [13] and differential protein synthesis as shown in other cells [14]. Strikingly, proteome analysis has revealed that structural and regulatory cytoskeletal proteins needed for cell shape change or migration are indeed upregulated in platelets [2]. Despite fast-growing information from proteomic analysis, cellular mechanisms for differential protein translation and cytoskeletal regulation are not fully understood.

In this work, we demonstrate that platelets that are exposed to different extracellular matrix components (collagen, laminin) or fibrinogen show a distinct phenotype and pattern of cytoskeletal arrangement after activation and that the translation initiation factor eIF4E characteristically redistributes to the ribosomal sites inside the cytoskeleton. eIF4E shows markedly enhanced mRNA binding and translation initiation that leads to increased synthesis of important cytoskeletal regulators such as vinculin, gelsolin, talin 1, filamin A, coronin or kindlin-3 (*FERMT3*). In this context, we investigated the effect of laminin on platelet protein synthesis for the first time. Our results underscore that soluble and matrix-based platelet activators result in a highly specific platelet phenotype formation by induction of a distinct translational response that generates a unique portfolio of functional proteins.

## 2. Results

### 2.1. De Novo Protein Synthesis of Activated Platelets Is Markedly Increased by Fibrinogen Binding

**Figure 1:**
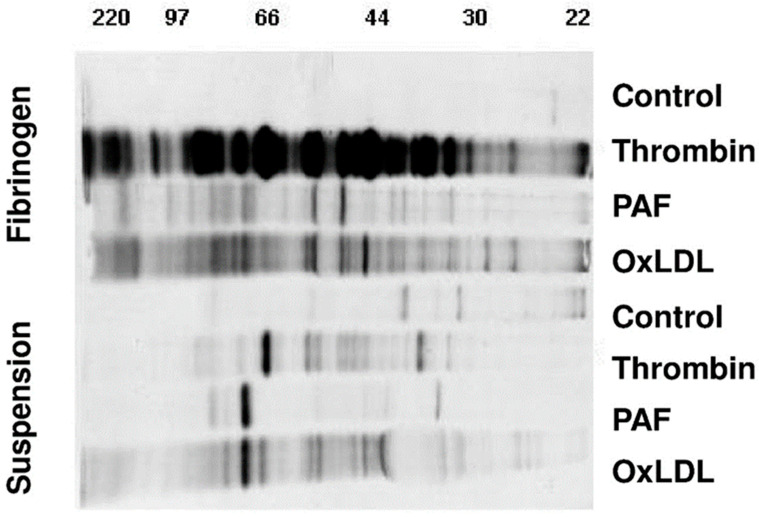
Protein de novo synthesis of fibrinogen-bound platelets is markedly increased compared to soluble platelets after thrombin activation.

Methionine is an essential amino acid for protein synthesis that is first incorporated into the newly forming peptides. To identify newly synthesized proteins, we added ^35^S-labeled methionine to the medium and incubated platelets together with the activators thrombin (0.1 U/mL), Platelet-activating factor (PAF, 1 nM) and oxLDL. Firstly, as shown in Figure 1, platelets synthesized a new set of proteins upon activation with thrombin, PAF or oxidized phospholipids (oxLDL), while quiescent platelets showed low baseline protein synthesis (control). Secondly, upon separation by SDS-PAGE, we observed that each platelet activator generated a specific protein panel depending on the stimulus (Figure 1). Thirdly, platelets adhering to fibrinogen-coated surfaces displayed a markedly increased ^35^S-methionine-labeled protein synthesis upon activation, especially with thrombin, compared to activated platelets without a fibrinogen matrix.

Washed platelets were incubated with ^35^S-Methionine to detect newly synthesized proteins for 18 h in the presence and absence of thrombin, PAF and oxidized phospholipids (OxLDL). Platelet proteins were separated by one-dimensional gel electrophoresis. Black bands display the differentially synthesized proteins during the 18 h incubation period. The four lower lanes show protein de novo synthesis in the absence, the above four lanes in the presence of a fibrinogen co-stimulus. Protein size markers ranging from 22 to 220 kDa are indicated above.

### 2.2. Adherent Platelet Morphology Is Specific to the Extracellular Matrix Stimulus

**Figure 2:**
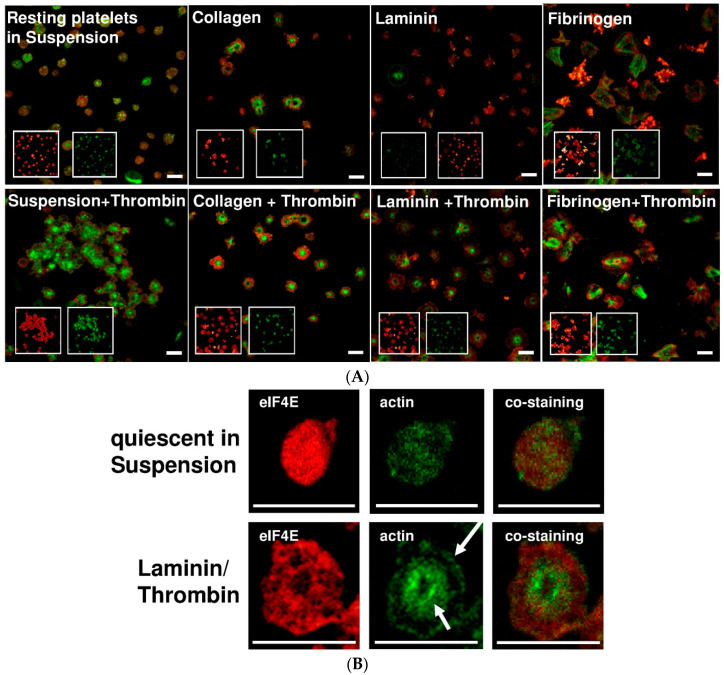
(**A**) Adherent stimulated platelets display a specific cytoskeletal morphology on a collagen, fibrinogen and laminin matrix with differential intracellular redistribution of eIF4E into the cytoskeleton. Platelets were incubated for 6 h in suspension or on collagen-, laminin- or fibrinogen-coated surfaces with and without thrombin activation. The platelets were fixed with paraformaldehyde and stained for actin (green) and eIF4E (red). The overlay panels show co-localization of eIF4E and actin and the different platelet phenotypes on the matrix surfaces. The inserts show actin or the eIF4E staining in separate channels. (**B**) Image magnification of quiescent and activated platelets on laminin surface stained for eIF4E (red) and actin (green) as well as eIF4E-actin overlay co-staining. White arrows exemplify areas of co-localization of eIF4E with the actin cytoskeleton. Scale bars represent 5 µm in Figure 2A,B.

To further investigate these phenotypic changes, platelets were adhered to the extracellular matrix surfaces collagen and laminin, to fibrinogen or were left in suspension. Each surface matrix generated a specific morphological pattern of adhesion. Additional activation with thrombin did not change the specific platelet morphology (Figure 2). Platelets were stained for the translation initiation factor eIF4E that is essential for the initiation of protein synthesis. As seen in Figure 2A, the different matrices generated a specific localization pattern for eIF4E (red) which showed a distinct redistribution into the cytoskeleton. The co-staining with actin visualized the platelet cytoskeleton and the individual cytoskeletal arrangement. The image magnification of quiescent and activated platelets on laminin in Figure 2B shows the initially diffuse localization of eIF4E and actin in a quiescent state and the characteristic phenotypic cytoskeletal rearrangement on laminin with redistribution and partial co-localization of eIF4E with the actin cytoskeleton (Figure 2B).

### 2.3. Platelet Activation Results in Polysomal Accumulation and Assembly of the Translational Machinery (Consisting of eIF4E, mRNAs and Vinculin)

**Figure 3:**
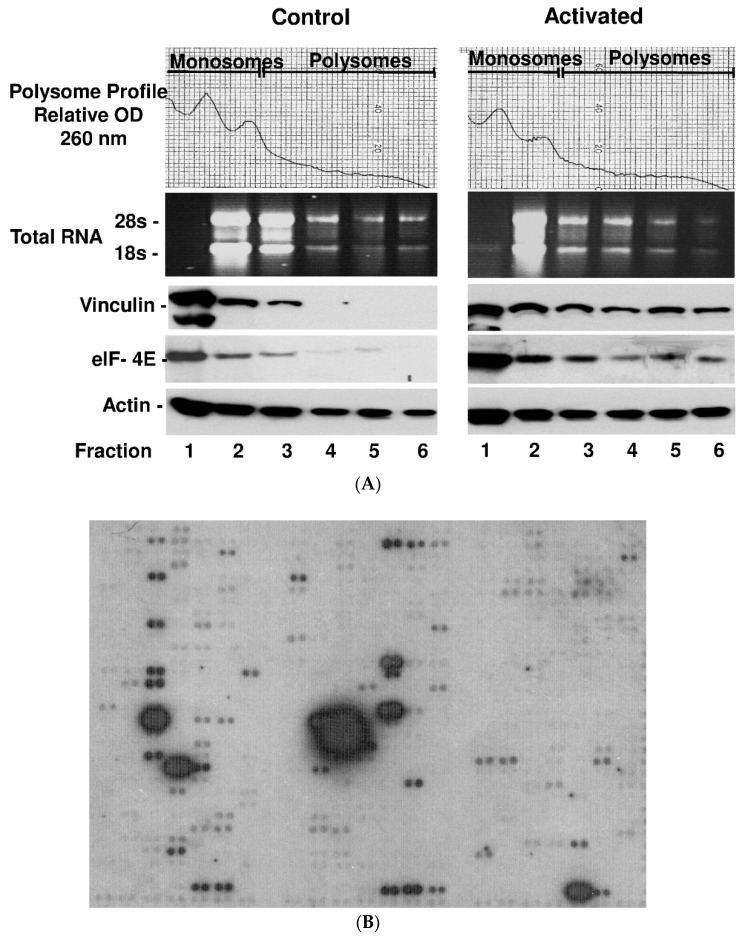
Platelet activation leads to polysomal assembly of mRNAs to translation factor eIF4E and polysomal accumulation of the cytoskeletal regulator vinculin. (**A**) Platelets were activated with thrombin for 5 min or left quiescent (control). Platelets were lysed and the polysome (lanes 4 to 6) and monosome fractions (lanes 1 to 3) were isolated. From these fractions, RNAs were separated on an agarose gel, the proteins by Western Blot analysis (lower three panels). In resting platelets, RNA, eIF4E and vinculin were primarily located in monosomes (lanes 1 to 3) and translocated and assembled in polysomes after thrombin activation (lanes 4 to 6). (**B**) From thrombin-activated platelets, the polysome fractions 4–6 were pooled and a cDNA array was performed that showed increased accumulation of several RNAs.

After binding of eIF4E to the mRNA cap, ribosomes run along the mRNAs and translate them into protein. Usually there are multiple ribosomes running along a mRNA strand (“polysomes”). We isolated the polysomal fraction of the ribosomes according to a standard protocol and found a significant accumulation of eIF4E and complexed RNAs as well as vinculin in polysomes. Monosome (lanes 1 to 3) and polysome (lanes 4 to 6) isolations of 3 independent samples before and after activation with thrombin are shown in Figure 3A. mRNA array analysis revealed that numerous other RNAs are recruited to the polysome fraction after activation (Figure 3B and Table S1). This recruitment to the polysomes is specific, since not all mRNAs can be found in the polysome fraction as shown in earlier experiments [15] and only represent a subfraction of total platelet mRNA. However, due to the limited range of the array-based mRNA analysis, we decided to focus on newly synthesized proteins in differentially activated platelets by a proteomics approach.

### 2.4. Platelets Show a Differential and Stimulus-Dependent Protein Expression Profile after Exposure to Extracellular Matrix Proteins Laminin or Collagen and to Fibrinogen

**Figure 4:**
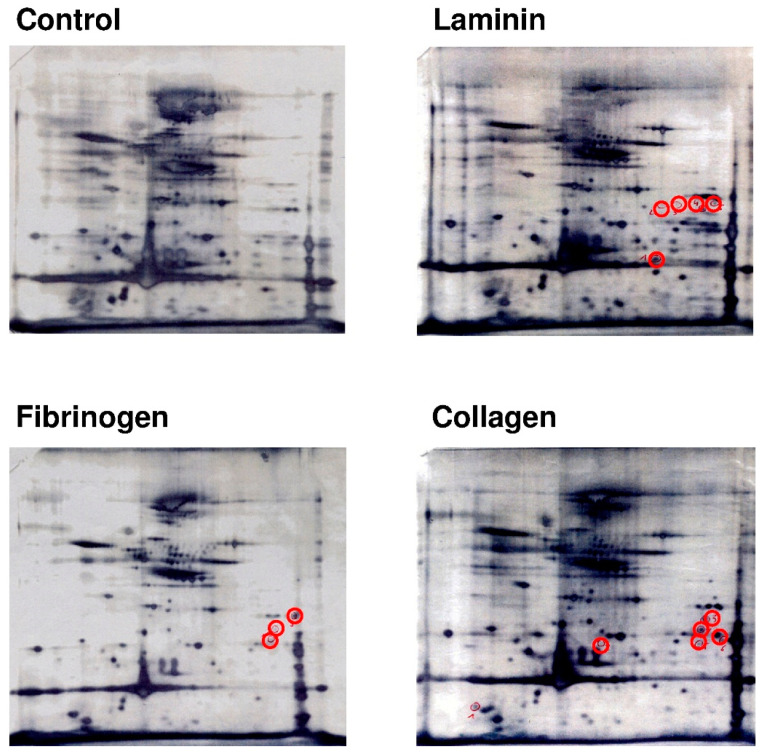
Platelet activation with collagen, fibrinogen or laminin leads to a stimulus-specific protein synthesis. Washed platelets were incubated on laminin, fibrinogen and collagen for 18 h. Platelets were lysed and proteins were separated by 2D-gel electrophoresis and visualized by silver staining. The red circles indicate a selection of proteins that are differentially expressed or upregulated on the different matrix surfaces compared to resting platelet controls.

As we first showed that de novo protein synthesis was increased in activated platelets adhering to extracellular matrix proteins, we now investigated the changes in the protein expression profile closer. Figure 4 shows the protein expression profile of platelets on laminin, fibrinogen and collagen surface compared to a quiescent platelet control. Stimulation with each of the extracellular matrix proteins resulted in a distinctly different pattern of protein expression in platelets (red circles).

### 2.5. Platelets That Are Adherent to Laminin Synthesize Proteins That Are Essential for Platelet Migration

**Figure 5:**
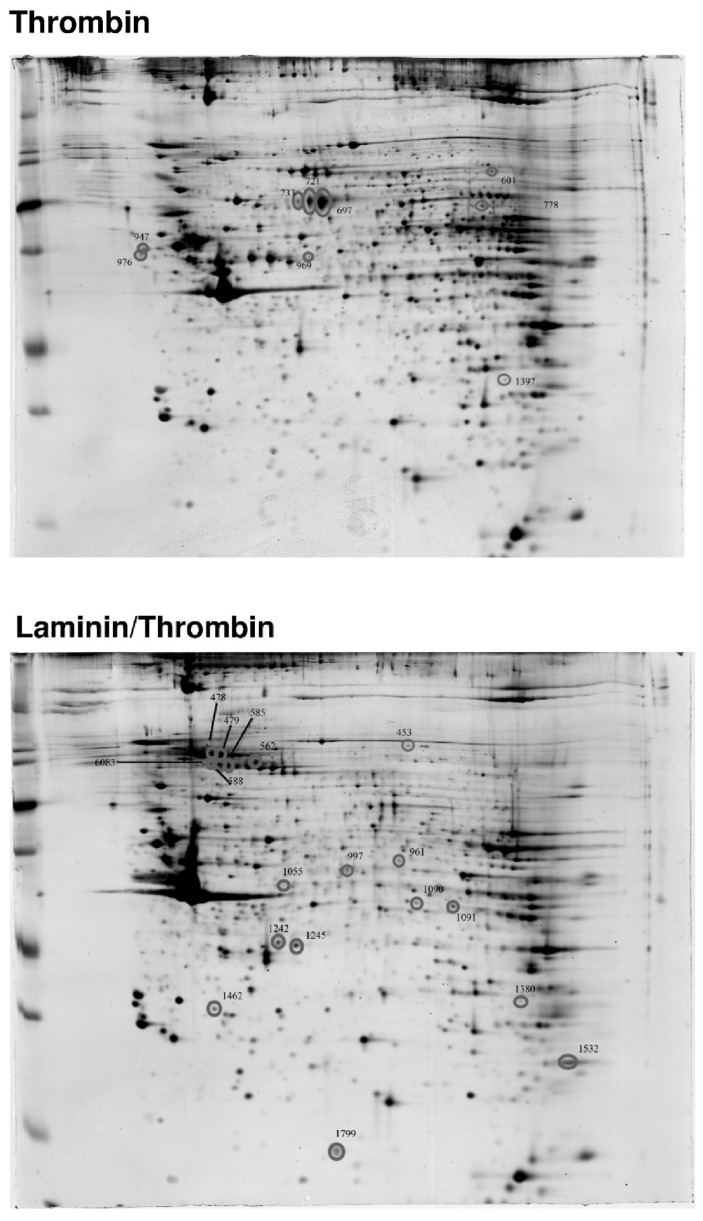
Platelet co-activation with laminin and thrombin results in synthesis of proteins that are essential for platelet motility.

To further identify proteins upregulated during platelet adhesion to extracellular matrix proteins, platelets were seeded onto laminin-coated surfaces and were additionally activated with thrombin for 18 h. Besides the novelty of data, our focus was set on laminin, because platelets can naturally be exposed to laminin from the basal membrane of blood vessels during vascular events. A high-resolution 2D-gel analysis was performed and the differentially expressed protein spots were excised, digested and protein fragments were analyzed by mass spectrometry (see numbered circles in Figure 5). Several important cytoskeletal proteins, such as actin, vinculin, talin-1, filamin A, gelsolin, coronin, kindlin-3 (*FERMT3*) and others, were upregulated after activation, most of which are especially involved in cytoskeletal rearrangement and the dynamic processes enabling cells to migrate. Corresponding Table 1 shows the results of the mass spectrometry analysis for each labeled protein spot by number. 

Washed platelets were incubated on laminin for 18 h and were additionally activated with thrombin at the start of the incubation (0.1 U/mL). Platelets were lysed and proteins were separated by 2D-gel electrophoresis and stained with flamingo red. The grey circles indicate proteins that were differentially expressed on laminin when compared to control (thrombin). Control platelets were activated with thrombin but kept in suspension. Differentially expressed proteins (numbered proteins spots #) were excised, digested and analyzed by mass spectrometry as listed in Table 1.

## 3. Discussion

The aim of this study was to explore differential platelet regulation and proteome changes of platelets on different extracellular matrix surfaces that may help to explain differences in hemostatic and migratory responses of platelets. Although numerous upregulated proteins have been identified in platelets after stimulation by thorough methods of proteomics [2], this has not been extended to intracellular reorganization processes in detail. Strikingly, most proteins that are upregulated or show differential regulation are structurally or functionally connected to the cytoskeleton and enable cytoskeletal rearrangement, motility and migration. By using methionine incorporation for detection of newly synthesized proteins, we confirmed up-front that the extent of protein synthesis markedly differs between platelet activators. Thrombin alone generated a comparable protein synthesis response in platelets in suspension as PAF or oxidized LDL, but markedly increased synthesis activity when platelets were additionally exposed to fibrinogen. Although all three activators showed increased protein synthesis with the fibrinogen co-stimulus, the effect was particularly pronounced for thrombin and it underscores the equal importance of the underlying adhesive matrix beyond activation. Previous work investigating the expression of BCL-3 has shown that co-stimulatory signals from fibrinogen and other platelet activators can modulate and increase selected protein synthesis [16]. Unfortunately, natural technical limitations occur with adherent, matrix-bound platelets, which prevented us from analyzing co-stimulatory effects of laminin/thrombin on newly synthesized proteins by the ^35^S-methionine incorporation method, which also does not allow protein spot sequencing.

Looking at an intracellular level, we observed that the adhesive phenotype of activated platelets was characteristically different when platelets were adherent to laminin, collagen or fibrinogen. For visualization of potential mechanisms for differential protein display, we were able to demonstrate that the translation factor eIF4E characteristically translocated to the cytoskeleton and partially co-localized with actin, which was phenotypically different on fibrinogen, collagen or laminin. Translation factors such as eIF4E, which we discuss in this article, aid in the assembly of ribosomes and mRNA to initialize protein synthesis. A regional intracellular translocation of the translational machinery during cell motility has also been observed in other cells [14]. We are convinced that the interesting observation of a stimulus- and surface-dependent translation factor redistribution likely explains why different activators generate a different set of proteins and a different platelet phenotype. We observed that a specific subset of mRNAs was transferred to the polysomes (Table S1), while we know from previous studies that a lot more of the existing mRNA is not translated [15]. Due to the limited range of the array-based mRNA analysis, we focused on the more comprehensive approach of differential protein synthesis in this study. Previous work from our own group and others showed that platelets display a different migratory phenotype on collagen as compared to fibrinogen [6,7]. Interestingly, we observed that platelets showed a trend to increased mobility on fibrinogen compared to collagen, which likely depends on the differential protein expression. Most importantly, integrins play a crucial role in the adhesion pattern that determines platelet function and integrin binding leads to an intracellular redistribution of mRNA and eIF4E [15,17]. Subsequently, 2D-gel analysis revealed that the proteome of platelets was markedly different in terms of protein upregulation when platelets were exposed to the different extracellular matrix proteins collagen and laminin or to fibrinogen.

In a comprehensive proteomic approach that compared protein expression in platelets with collagen, thrombin and arachidonic acid on a larger scale, Majek and colleagues were able to identify 144 proteins that were upregulated in platelets after activation [2]. They show that protein expression was dependent upon the platelet activator both in terms of the amount of protein and the distribution of proteins, which impressively supports the concept of differential protein synthesis. Although the effect of platelet activation by laminin with or without co-stimulus was not investigated in previous studies, strikingly, the analysis showed especially an upregulation of numerous cytoskeletal proteins and proteins that regulate protein translation, underscoring the importance of specialized protein synthesis for cytoskeletal regulation. Regulation of platelet functions on a protein level, however, not only occurs by de novo protein synthesis but includes post-transcriptional modification such as phosphorylation/dephosphorylation [18] or proteolytic cleavage [19]. To investigate how each of the identified proteomic changes occurs and whether the protein identified is newly synthesized or a cleavage product will have to be gradually answered by future work. Indeed, previous work has demonstrated a characteristic protein cleavage of the cytoskeletal proteins talin-1 and filamin A during collagen activation of platelets as a likely mode of rapid protein modification [20]. In additional work, we were able to demonstrate that the proteasome is activated during collagen-induced platelet aggregation and that filamin A and talin 1 are cleaved by the proteasome in the same characteristic fashion [21]. In patients with myocardial infarction, we were able to demonstrate that regulators of the cytoskeleton such as kindlin-3 are modified by proteolytic cleavage in platelets as well [22]. We previously identified that heat shock protein 27 (HSP27) upregulation also plays a role in myocardial infarction [23] and interestingly HSP27 mRNA was found in the polysomal RNA fraction of activated platelets in this study as well (Figure 3B and Table S1). Cleavage of cytoskeletal proteins such as talin and filamin by calpain, for instance, has also been discovered as a mechanism to regulate migration activity [13]. Focusing on protein synthesis, exciting discoveries of the previous years have revealed how anucleate platelets perform protein synthesis [1,24,25,26]. Besides transcription from inherited, pre-formed mRNA from megakaryocytes [27], platelets also have the capacity to alternatively splice RNA after activation [1] and there is evidence that regulation also occurs on a superior level by miRNA [28]. Using the platelet activators collagen and TRAP, Nassa and colleagues [3] were able to demonstrate that a large pool of mRNA is spliced after activation and that several proteins that regulate cytoskeletal or migratory processes are increasingly synthesized. Consistently, by precipitation of eIF4E, we observed a significant increase of mRNA binding to translation initiation factor eIF4E (Figure S1) and in polysomes (Figure 3B) after activation, suggesting a comprehensive activation of the translation machinery and protein synthesis after activation. eIF4E characteristically accumulated in the polysomes as shown above and we observed increased protein levels of important cytoskeletal regulators such as vinculin. Vinculin plays an important role in the structural organization of active fibers and is thus involved in cytoskeletal regulation [29]. During our studies investigating the phenomenon of platelet migration, we observed intracellular translocation of vinculin into focal adhesion complexes in migrating platelets [7] and previous work has speculated that vinculin determines cellular mobility by stabilizing actin fibers. 

This study now further revealed that a whole set of proteins with important biologic relevance for cytoskeletal dynamics such as vinculin, gelsolin, filamin A, talin 1, kindlin-3 (*FERMT3*) or coronin are upregulated after activation with laminin. For the first time, our study thus contributes insight into the proteomic phenotype of laminin-activated platelets that has not been investigated so far. We focused on laminin because platelets are exposed to laminin during acute vascular events and likely encounter laminin in vessels with endothelial damage, where platelet migration may execute important biological functions. As previously shown, migration allows platelets to fight invasive pathogens and to enter the vessel wall [5,6,7]. As mentioned above, the cytoskeletal proteins talin 1 and filamin A are regulated in response to platelet activity and aggregation, so it is not surprising that particularly cytoskeletal proteins are synthesized in a stimulus-specific manner during activation. Although we focused on proteins that are increasingly synthesized after activation, protein secretion and vesiculation from platelets is another important biological event that affects the intracellular set of platelet proteins besides the proteomic regulation mechanisms described above. Platelets store a huge variety of vasoactivate proteins in preformed vesicles which are rapidly released with activation and which may then be lost for analysis and change the proteome of activated platelets [30,31,32,33]. All proteins that we discuss in this study have been shown to be involved in the regulation of cell migration and mobility: gelsolin is critical for actin assembly/disassembly and podosome formation [34], vinculin for focal adhesion dynamics [35], coronin for dynamic F-actin binding [36], kindlin-3 (*FERMT3*) [37] and talin 1 are critical in signal transfer between integrins and the cytoskeleton, while filamin A [38] is essential for the interaction between membranes and actin fibers. Certainly the need for a finely coordinated cytoskeletal rearrangement is different during clot formation or migratory action, so it appears even more likely that activation of the required proteins depends on the task, environment and stimulus for an adequate individual cellular response.

The ^35^S-methionine incorporation method that would best identify newly synthesized proteins cannot be applied to matrix-bound platelets on laminin, especially not in combination with 2D-gel electrophoresis and protein sequencing by mass spectrometry, due to natural limitations of the method. We therefore chose a comparative conventional 2D-gel analysis with protein sequencing from platelets activated with laminin/thrombin compared to thrombin control to demonstrate the effect of laminin towards a differential upregulation and increase of selected proteins. 

Limitations of this study further arise from the technical inability to isolate and analyze polysomal mRNAs in a stimulus-specific manner from platelets adherent to matrix, which prevented us from an analysis of laminin-specific mRNA translation from polysomes. Due to the complexity of platelet proteome regulation on different cellular levels, unequalizable experimental conditions and natural technical limitations of the polysome analysis method as discussed above, polysomal mRNAs that we identified by general platelet activation merely overlap with the differentially increased proteins identified by mass spectrometry, which makes it impossible to prove a run-through mechanism of our findings at this stage. 

Showing a stimulus-dependent differential intracellular reallocation of eIF4E, differential protein upregulation on different extracellular matrices, including the first analysis of platelets on laminin, and increase of critical cytoskeletal proteins such as vinculin, gelsolin, talin-1, filamin A, coronin or kindlin-3, our work adds new insight and supports the concept of a differential activation-dependent platelet protein synthesis. Although we did not investigate migration itself, this work may help to improve our understanding of activation-dependent protein translational responses on platelets during mobility and for future studies.

## 4. Material and Methods

### 4.1. Platelet Isolation

Washed platelets were isolated from acid-citrate-dextrose–anticoagulated human blood (approved by the local ethics committee of the University of Tuebingen, No. 264/2007BO2 on 25 October 2007) as described previously [39]. Platelets (5 × 10^8^ total) were resuspended in M199 and were left quiescent in suspension or adhered to fibrinogen (100 mg/mL), collagen (500 ug/mL) or laminin-coated surfaces (100 μg/mL) in 6 well plates for 6–18 h. In some indicated experiments, co-incubation occurred with an additional amount of fibrinogen (100 µg/mL). The platelets were lysed in 0.5 mL of RIPA buffer until further use or fixed with 0.5% paraformaldehyde on each surface and blocked with 0.5% bovine serum albumin for confocal microscopy.

### 4.2. Metabolic Radiolabeling of Human Platelet Proteins

Platelets (1 × 10^9^ total) were placed in 1 mL of methionine-free M199 medium for 2 h followed by the addition of 50 µCi (1 Ci = 37 GBq) of [^35^S]methionine (GE Healthcare, Wauwatosa, WI, USA)) for each sample. After the addition of labeled methionine, the platelets were left in solution without any activator for 30 min. Then they were activated with thrombin, PAF or oxidized phospholipids in the presence of fibrinogen or not, or left quiescent without any activator (= control). Platelets were gently rocked and six hours later, the platelets were washed with Tris-buffer, the suspensions were centrifugated and the supernatants were removed. The cell pellets were lysed in RIPA-buffer on ice and identical volumes of the platelet lysates were separated by SDS-gel electrophoresis on a 9% gel. The gels were dried for 2 h in an 80 °C vacuum oven and exposed overnight to Kodak MS film placed in a −80 °C refrigerator.

### 4.3. Two-Dimensional Electrophoresis

Platelets exposed to the extracellular matrix proteins laminin and collagen or fibrinogen or quiescent platelets controls were lysed in 100 µL of CHAPS lysis buffer (8 M Urea, 4% CHAPS, and 2% DTT) and purified using a 2D clean-up kit (GE Healthcare, Freiburg, Germany) according to the manufacturer’s instructions. Comparative 2D gel analysis of the proteomes was performed as described previously with slight modifications [40,41]. First dimension isoelectric focusing was performed using a Protean IEF cell focusing unit (BioRad, Hercules, CA, USA) with pH 3–10 NL gel strips (11 to 28 cm, depending on experiment, GE Healthcare, for some subsequent gels pH 4–7). For the second dimension equilibrated (5.7 M Dithiotreitol and 1.5 M Iodoacetamide) gel strips were applied to 12% polyacrylamide gels. Proteins were stained with silver, lava purple or flamingo red fluorescent dye (Fluorotechnics, Sydney, Australia). Protein spots of interest were excised from the gels using a spot picker, digested with trypsin and analyzed by LC ESI-MS/MS (Applied Biosystems/MDS Sciex, Darmstadt, Germany) as described previously [40]. Some 2D gels and some one-dimensional SDS-PAGEs, platelet proteins were incubated with [^35^S]methionine for 8 h to identify newly synthesized proteins. These ^35^S-labeled proteins were separated by 2D-gel analysis as described above. These gels were dried in a vacuum oven and then exposed to Kodak film at −80 °C overnight.

### 4.4. Immunoblotting

Platelets lysed in Laemmli buffer were separated by SDS PAGE gel electrophoresis and probed for eIF-4E, vinculin and β-actin. After protein transfer to a nitrocellulose membrane, membranes were blocked in 5% non-fat milk in Tris-buffered saline with Tween-20 (TBS-T, Sigma-Aldrich, Taufkirchen, Germany) for 1 h. Afterwards, primary monoclonal antibodies were added. eIF4E, actin and vinculin were detected using specific mouse anti-human antibodies (1:1000) (anti-eIF4E, BD Transduction Laboratories, San Diego, CA, USA; anti-actin, ICN Biomedicals, Costa Mesa, CA, USA; anti-vinculin, Upstate Biotechnology, Placid, NY, USA) and incubated overnight at 4 °C with constant agitation. Membranes were washed in TBS-T repeatedly, and HRP-conjugated secondary antibody (1:10,000) (GE Healthcare, Freiburg, Germany) was added for 1 h at room temperature. After washing, chemiluminescent substrate (ECL reagent, Amersham Biosciences, Munich, Germany) was added for 1–5 min and bands were visualized on plain film. β-Actin served as loading control (1:1000, rabbit anti-human β-Actin, Cell Signaling Technology, Dallas, TX, USA).

### 4.5. Confocal Microscopy

Immunofluorescence staining was performed as previously described [7]. In brief, washed platelets were allowed to adhere to a fibrinogen, collagen or laminin surface (50 μg/mL) on a chamber slide for 20 min with and without thrombin activation (0.1 U/mL) and were then fixed with paraformaldehyde (2%) and permeabilized with Triton-X-100 (TX-100, 0.025%). The adherent platelets were washed and blocked with 2% bovine serum albumin for 30 min followed by incubation with the primary antibody for 2 h at room temperature. Primary antibody against eIF4E (Cell Signaling Technology, Dallas, TX, USA) was used in a 1:100 dilution in TBS with 1% BSA (Bovine Serum Albumine). Slides were then washed and incubated with an Alexa 594-linked secondary antibody (Dianova, Hamburg, Germany) for 1 h. For Actin, we stained with Oregon-green-(488) coupled phalloidin. Confocal microscopy was performed using a Zeiss LSM 5 EXCITER confocal laser scanning microscope (Carl Zeiss Micro Imaging, Jena, Germany).

### 4.6. Polysome Analysis

Polysomes were isolated from platelets using minor modifications of published protocols [42]. In brief, platelets (7.5 × 10^9^) were lysed in a high-salt buffer to remove polysomes from the cytoskeleton (200 mM Tris, 520 mM KCl, 30 mM MgCl_2_, 4% Triton X-100, and 200 mM sucrose, pH 9.5). The cells were passed three times through a 21-gauge tuberculin syringe, followed by a brief centrifugation to remove all insoluble material and heparin (10 mg/mL), and NaCl (150 mM) was added to the lysate. Mitochondria were removed by further centrifugation (5 min, 14,000 g) and the resulting supernatant was placed on a 4-mL sucrose gradient (0.5–2 M) and centrifuged at 4 °C for 2 h (43,700 rpm) using an SW55 Ti swinging bucket rotor (Beckman Coulter). Sucrose gradients were fractionated using an ISCO UA-6 254-nm continuous flow chamber into 760 µL aliquots. Total RNA in each fraction was isolated for analysis of ribosomal RNA and mRNA from fractions 4–6 was pooled for array analysis (array 7742-1; CLONTECH Laboratories, Inc. (Mountain View, CA, USA)).

### 4.7. cDNA Arrays

Total RNA was extracted from the polysome fraction as described above. Five µg of RNA was converted to 32P-labeled first strand cDNA using Superscript Reverse Transcriptase (Life Technologies, Inc.) and a primer mix obtained from CLONTECH (Palo Alto, CA, USA). The labeled probe mix was subsequently hybridized to atlas array number 7742-1 (Clontech) as described by the manufacturer. In brief, hybridization was performed at 68 °C overnight in a rotating chamber and washed three times in a TBS-T buffer for 20 min, dried and then exposed to Kodak MS film.

## Figures and Tables

**Table 1:** Differentially expressed proteins in platelets after exposure to laminin, identified and sequenced by mass spectrometry.

Protein Spot #	Protein Identified
spot #1055	Tubulin beta-1 chain
spot #1090	Isoform 2 of gelsolin
spot #1091	Isoform 2 of gelsolin
spot #1242	FERMT3 Isoform 2 of Fermitin family homolog 3
spot #1245	FERMT3 Isoform 2 of Fermitin family homolog
	Transaldolase (TALDO1)
spot #1462	CAPZB cDNA FLJ60094, highly similar to F-actin
	capping protein subunit beta
spot #1532	FGA Isoform 1 of Fibrinogen alpha chain
spot #1799	CAP1 Isoform 1 of Adenylyl cyclase-associated protein1
	HK1 Isoform 3 of Hexokinase-1
spot #478	VCL Isoform 1 of Vinculin
	TLN1 Talin-1
	ACTN1 Alpha-actinin-1
	FGG Isoform Gamma-B of Fibrinogen gamma chain
	ACTN4 Alpha-actinin-4
spot #479	VCL Isoform 1 of Vinculin
	TLN1 Talin-1
	ACTN1 Alpha-actinin-1
	FGG Isoform Gamma-B of Fibrinogen gamma chain
	ACTN4 Alpha-actinin-4
spot #562	TLN1 Talin-1
	FLNA Isoform 2 of Filamin-A
	TLN2 Talin-2
spot #585	VCL Isoform 1 of Vinculin
	TLN1 Talin-1
	GSN Isoform 2 of Gelsolin
spot #588	VCL Isoform 1 of Vinculin
	GSN Isoform 2 of Gelsolin
spot #6083	VCL Isoform 1 of Vinculin
spot #961	CORO1C Coronin-1C
spot #997	CORO1A Coronin-1A
	ENO1 Isoform alpha-enolase of alpha-enolase

## Supplementary Materials

The following are available online at https://www.mdpi.com/1422-0067/21/21/8155/s1, Table S1: mRNAs identified in polysomes of thrombin-activated platelets. Figure S1: Overview of mRNA bound to precipitated translation initiation factor eIF4E in quiescent and activated platelets.
