# Supplementary material for: Extracellular Matrix-Specific Platelet Activation Leads to a Differential Translational Response and Protein De Novo Synthesis in Human Platelets
Source: Int J Mol Sci. 2020 Oct 31;21(21):8155. doi: 10.3390/ijms21218155 (PMC7672557; doi:10.3390/ijms21218155)
Supplement: Supplementary file 1 [file ijms-21-08155-s001.zip › ijms-963414-supplementary.docx]

**Supplemental Figure S1: Overview of mRNA bound to precipitated translation initiation factor eIF4E in quiescent and activated platelets**

**A**


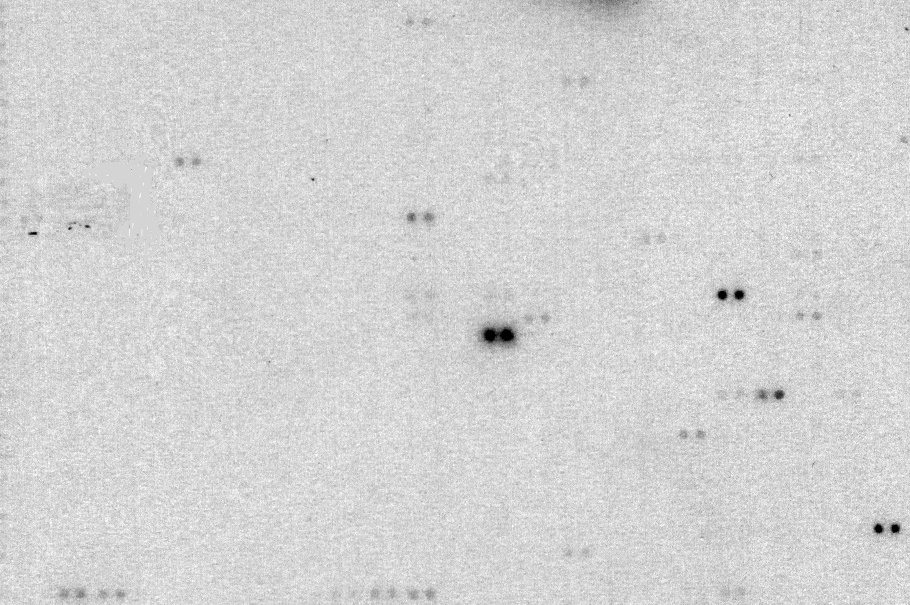


Quiescent platelets

**B**


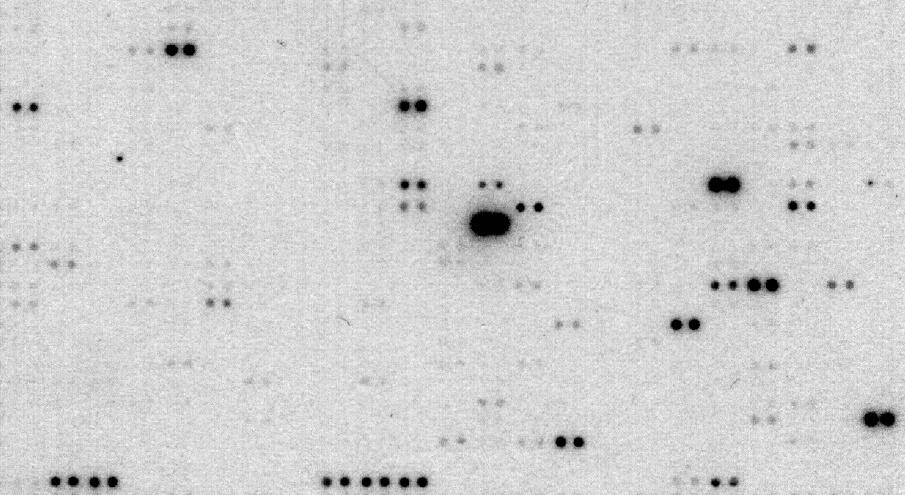


Thrombin-activated platelets

**Supplemental Figure S1:** mRNA bound to eIF4E was isolated by precipitation of eIF4E and bound mRNA was visualized by cDNA array in quiescent (**A**) and thrombin-activated platelets (**B**). mRNA binding to eIF4E was markedly increased when platelets were activated while only few mRNAs are bound to eIF4E under unstimulated conditions.

###### Supplemental Table S1. mRNAs identified in polysomes of thrombin-activated platelets

Heat shock protein (HSP) 27

Rho GDI beta

Ubiquitin

Ubiquitin conjugating enzyme

Gluthathione S transferase

Osteonectin

Semaphorin, CD100

TIMP-1

Growth factor receptor-bound protein-2

Nerve growth factor receptor

Platelet basic protein precursor

GAPDH

p21-rac1

Tubulin alpha 1 subunit

Cyclin-dependent kinase inhibitor 1

PDGFA subunit precursor

GPIIIa

CD9 antigen

Basigin precursor

Beta actin

Cadherin 4

IL-1beta precursor

23 kda highly basic protein

Vimentin

Integrin-linked kinase

CDC10 protein homolog
